# Cannabidiol Strengthening of Gastric Tight Junction Complexes Analyzed in an Improved *Xenopus* Oocyte Assay

**DOI:** 10.3390/membranes14010018

**Published:** 2024-01-08

**Authors:** Laura Stein, Marie-Luise Vollstaedt, Salah Amasheh

**Affiliations:** Institute of Veterinary Physiology, School of Veterinary Medicine, Freie Universität Berlin, 14163 Berlin, Germany; laura.stein@fu-berlin.de (L.S.); marie-luise.vollstaedt@fu-berlin.de (M.-L.V.)

**Keywords:** claudins, epithelial barrier, *Xenopus* oocytes, tight junction, heterologous expression, stomach

## Abstract

Cannabidiol (CBD), the non-psychoactive compound derived from the cannabis plant, has gained attention in recent years as a remedy against gastrointestinal disorders ranging from nausea and inflammation to abdominal pain. Recent advances demonstrated an effect on inflammatory pathways and barrier proteins. However, information on possible direct effects is scarce and needs to be addressed, as applications are currently increasing in popularity. To accomplish this, we have employed *Xenopus laevis* oocytes as a heterologous expression system for analysis of the direct effects on stomach-specific claudins and further developed tight junction (TJ) protein interaction assays. Human claudin-4, claudin-5, and claudin-18.2 were expressed in *Xenopus* oocytes, clustered in pairs to form contact areas, and analyzed in a two-cell model approach, including measurement of the contact area and contact strength. CLDN4/5/18 + CLDN4/5/18 oocyte pairs were incubated with 20 µM CBD or with 40 µM CBD and were compared to cells without CBD treatment (ctrl). For interaction analysis, the contact area was measured after 24 h and 48 h. Whereas CBD did not affect the size of the protein interaction area, Double Orbital Challenge experiments revealed an increased contact strength after 24 h incubation with CBD. In addition, the *Xenopus* oocyte experiments were accompanied by an analysis of claudin-4, -5, and -18 expression in gastric epithelium by immunoblotting and immunohistochemistry. Claudin-4, -5, and -18 were strongly expressed, indicating a major role for gastric epithelial barrier function. In summary, our study shows direct effects of 40 µM CBD on Xenopus oocytes heterologously expressing a stomach-specific claudin combination, indicating a supportive and beneficial effect of CBD on gastric TJ proteins.

## 1. Introduction

*Xenopus laevis* oocytes have been established as a valuable heterologous expression system for the functional characterization of membrane proteins [1]. This research model is still evolving because of its distinctive features, as the large cells allow manipulation including the microinjection of nucleic acids and function as an *in vivo* translation system due to the high translational capacity [2,3]. The *Xenopus* oocyte therefore represents a unique expression system alongside cell-culture or tissue models for analyzing proteins. In particular, membrane transporter analyses combined with electrophysiological techniques in single-cell approaches have benefitted from this model [4,5,6]. Performing interaction studies by pairing oocytes with heterologously expressed gap junction proteins such as connexins or tight junction (TJ) proteins such as claudins extended the possibilities of using *Xenopus* oocytes, providing important insight into the physiological function and composition of protein complexes [7,8,9,10,11,12]. Moreover, the oocyte is capable of tolerating different physical and chemical environments for analyses, e.g., in acidic pH ranges [13], and it endogenously expresses the scaffolding protein ZO-1 regarded as a prerequisite for proper membrane insertion and functionality [14].

Claudins are tissue-specific and expressed in epithelial cells. Their interactions are based on head-to-head connections via the extracellular loops between adjacent cells, thus making a major contribution to TJ assembly and claudin integrity, which affects organ-specific barrier properties. This is based on claudin strand arrangement due to homo- or heterophilic interactions and by building paracellular networks via *trans*-interaction adjusting the permeability for molecules [15,16,17].

The intact gastric epithelial is described as a very tight epithelium [18], expressing primarily claudin-4, -5, and -18 [19,20,21,22]. The paracellular gastric barrier is based on the TJ assembly of these claudins, which have to prevent the diffusion of ions, digestive enzymes, toxins, food substances, microbiota, pharmaceuticals, and other xenobiotics [18], and noxious substances in luminal chyme or bacteria as *Helicobacter pylori* can damage the gastric mucosa and epithelial cells, resulting in an altered claudin expression. Furthermore, the perturbation of the gastric mucus can cause acidic pH contact to the epithelial cells, although normally, the pH of epithelial surface cells is almost neutral [23]. These alterations may lead to the loss of several functions and increase the risk for many gastric diseases such as gastritis and stomach cancer [24].

Thus, an intact stomach barrier is crucial to address the number of essential tasks in a complex environment with constantly changing physical and mechanical challenges.

As claudins represent the primary structural correlate for gastric epithelial barrier function, TJ-modulating substances like secondary plant compounds have attracted interest in improving gastric epithelial barrier integrity.

Cannabidiol (CBD), as the major non-psychoactive phytocannabinoid component of the herbal *Cannabis sativa* L., has been shown to have beneficial, anti-inflammation effects including positive effects on epithelial barrier integrity. This mechanism was analyzed in the non-transformed porcine intestinal epithelial cell line IPEC-J2 by our group recently, including the observation of an enhancement of barrier protein claudin-4 expression [25].

Previous studies of tight junction proteins in the gastrointestinal system have largely focused on the colon and large intestine, and not much is known about the claudin assembly in the stomach. Our goal was to examine the effects of CBD on claudins of the sealing gastric epithelium. We hypothesized that CBD may have a direct strengthening effect on the gastric TJ protein *trans*-interaction of human claudin-4, -5, and -18. To test this hypothesis, we used *Xenopus* oocytes as a heterologous expression and interaction system and functionally characterized the major gastric TJ proteins as co-expressed gene products by investigating the *trans*-interaction properties: contact area size and contact strength of claudin-4, -5, and -18 expressing and paired oocytes.

## 2. Materials and Methods

### 2.1. Animals

Care and treatments of *Xenopus laevis* are subjected to the German Animal Welfare Act regulations and were admitted by the animal officer for the Freie Universität Berlin and the governance of the State Office for Health and Social Affairs (Landesamt für Gesundheit und Soziales Berlin, Germany, permit E 0061/23).

### 2.2. Chemicals

First, 10 mM cannabidiol (CBD; Tocris, Bristol, UK) stocks were prepared and were stored at −20 °C. Directly before the CBD treatment for oocytes started, stocks were diluted with oocyte Ringer (ORi) to result in concentrations of 20 μM and 40 μM.

### 2.3. Oocyte Harvesting

A surgical laparotomy was carried out to harvest unfertilized oocytes from female *Xenopus laevis.* To accomplish this, animals were anesthetized in a 1 L bath containing buffered 0.2% tricaine (ethyl 3-aminobenzoate methanesulfonate, Sigma–Aldrich, Taufkirchen, Germany) for 5–10 min at room temperature.

The duration of the bath was kept animal-specific, and anesthetic depth was controlled by testing the loss of righting and corneal reflexes.

The ovarian lobes in the abdominal cavity were removed with sterile forceps after making a small skin and muscle incision. Afterward, the connective tissue strands of the ovarian tissues were digested for 90 min by a solution of 1.5 mg/mL collagenase solved in oocyte Ringer (ORi), and follicular cells were removed for 10 min in Ca^2+^-free ORi on a mechanical shaker.

Only high-quality oocytes of the maturity stages V and VI (diameter > 1000 μm) were selected by optical inspection and were covered with fresh oocyte Ringer (ORi) for microinjection as described previously [10].

### 2.4. cRNA Preparation and Injection

The cRNAs coding for CLDN4, CLDN5, and CLDN18.2 (Lot. No. 1989156, Lot. 1989155, No Lot. No. 25442, Life Technologies, Carlsbad, CA, USA) were synthesized by cloning each human nucleotide coding sequence into a high copy ampicillin-resistant pGEM (vector for transformation in competent DH10b *Escherichia coli*, as reported recently) [13,14]. *In vitro* transcription was carried out with a T7 RNA-polymerase-based transcription system (T7 RiboMAX RNA Production System, Ribo m7G Cap Analog, Promega, Walldorf, Germany). cRNA sizes were verified by gel electrophoresis, and concentration and purity were measured by UV spectroscopy (Nanodrop, P330, Implen, München, Deutschland). cRNA represents the synthetic transcript of each claudin DNA, synthesized by an *in vitro* transcription system, as described above.

One day after oocyte harvest, cRNA encoding claudin-4, claudin-5, and claudin-18 (CLDN4/5/18) were injected (Nanoliter 2010, World Precision Instruments, Sarasota, FL, USA) into each oocyte. The cRNA amount of each claudin was 1 ng per oocyte in a total injection volume of 50.6 nl and a cRNA concentration of 20 ng/μL.

RNase-free water-injected cells served as control (ctrl) oocytes. For microinjection, cRNA or RNase-free water was filled in a glass capillary manufactured by a horizontal puller (P-97 Micropipette Puller, program 11, Sutter Instrument Company, Novato, CA, USA), and the oocytes were placed in a plexiglass plate with milled rows.

After 3 days of expression at 16 °C, claudin interaction analyses were started.

### 2.5. Protein Extraction of the Oocyte Membrane Fractions

Protein preparations of pooled membrane fractions of ten oocytes were used for immunoblotting. To accomplish this, oocytes were suspended in 500 μL homogenization buffer (MgCl_2_ (5 mM), NaH_2_PO_4_ (5 mM), EDTA (ethylenediaminetetraacetic acid) (1 mM), sucrose (80 mM), and Tris (Tris (hydroxymethyl) aminomethane) (20 mM); pH 7.4). After two centrifugation steps at 200 rpm for 10 min at 4 °C (Sigma 3–30KS, Sigma-Aldrich, Munich, Germany), cell debris was discarded. Next, the supernatant was centrifuged once at 13,000 rpm for 30 min at 4 °C. The resulting pellet contained the cell membrane fractions and was resuspended in 80 to 150 μL homogenization buffer depending on the protein concentration.

The protein concentration was determined by a Pierce 660 nm Protein Assay (Thermo Fisher Scientific, Hennigsdorf, Germany) in a 96-well plate using a 562 nm plate reader (PerkinElmer EnSpire Multimode Plate Reader, Waltham, MA, USA) with bovine serum albumin standards (Thermo Fisher Scientific, Hennigsdorf, Germany).

### 2.6. Protein Extraction of Stomach Tissue

Stomach tissue samples (fundus) were obtained from the slaughterhouse, and samples were frozen in liquid nitrogen and stored at −80 °C. For immunoblotting, samples were homogenized in RIPA buffer (HEPES (25 μM, pH 7.6), NaF (25 μM), EDTA (2 M), 1% SDS (10%), HO, and enzymatic protease inhibitors (Complete EDTA-free, Boehringer, Mannheim, Germany)). After a centrifugation step for 1 min at 16,000× *g* at 4 °C (Sigma 3–30KS, Sigma-Aldrich, Munich, Germany), the supernatant was transferred for lysis on ice for 30 min. Subsequently, the samples were centrifuged for 15 min at 15,000× *g* at 4 °C.

The stomach proteins were quantified after pooling the samples of three animals by a Bio-Rad DC Protein Assay (Bio-Rad Laboratories GmbH, Munich, Germany) in a 96-well plate using a plate reader (PerkinElmer EnSpire Multimode Plate Reader, Waltham, MA, USA) with bovine serum albumin standards (Thermo Fisher Scientific, Hennigsdorf, Germany).

### 2.7. Immunoblotting of Oocyte Membrane Fractions and Stomach Tissue

Immunoblotting of oocyte membrane fractions and stomach tissue was carried out by a stain-free immunoblotting kit (Stain Free TGX, Fast Cast Acrylamide, Bio-Rad, München, Deutschland). To prepare the samples for immunoblotting, 4 × Laemmli (Bio-Rad Laboratories, Munich, Germany) was added to all samples. After denaturation, samples were loaded onto a 10% SDS polyacrylamide gel. Proteins were transferred to the PVDF membrane, and 5% non-fat dry milk in Tris-buffered saline was used for blocking unbound membrane sites to prevent the non-specific binding of the antibodies.

Specific primary antibodies against claudin-4, claudin-5, and claudin-18 (Invitrogen #32-9400, #34-1600, #700178, Life Technologies, Carlsbad, CA, USA) and peroxidase-conjugated goat anti-rabbit and anti-mouse antibodies (#7074, #7076, Cell Signaling Technology, Danvers, MA, USA) were used for protein detection by a ChemiDoc MP system (Bio-Rad Laboratories) after adding detection solution (Clarity Western ECL Blotting Substrate, #1705061, Bio-Rad Laboratories GmbH, Munich, Germany). As controls (ctrl), oocytes injected with RNase-free water were employed.

### 2.8. Immunohistochemistry of Oocytes and Porcine Stomach Tissue

For immunohistochemistry, injected oocytes were fixed overnight at 4 °C in 4% PFA (16% paraformaldehyde, E15700, Science Service, Munich, Germany). Via a 70% ethanol to xylol gradient, oocytes and stomach tissue (fundus) were dehydrated followed by embedding cells in paraffin.

Cross-sections (5 μm) of oocytes and stomach tissue were first rehydrated in a xylol-ethanol gradient. Next, slides were boiled for 30 min (oocytes) and 45 min (stomach tissue) in citrate buffer (pH 4). After a permeabilization step for 5 min at room temperature with Triton X-100 in PBS +/+, samples were framed with a hydrophobic PAP pen (Kisker Biotech GmbH & Co. KG, Steinfurt, Germany).

The sections were then permeabilized for 5 min at room temperature in Triton X-100 in PBS +/+ followed by an oocyte and stomach tissue framing step using a PAP pen (Kisker Biotech GmbH & Co. KG, Steinfurt, Germany). Specific primary claudin-4, claudin-5, and claudin-18.2 antibodies (Invitrogen #32-9400, #35-2500, #34-1600, #700178, Life Technologies, Carlsbad, CA, USA) were added for 1 h at 37 °C after blocking the sections (5% goat serum and 1% bovine serum in PBS +/+). After washing out the primary antibodies with blocking solution, specific secondary goat anti-rabbit Alexa Fluor-488 and goat anti-mouse Alexa Fluor-594 were incubated overnight at 4 °C. Secondary antibodies were washed out with blocking solution and distilled water, and the samples were mounted in ProTaqs Mount (Flour Biocyc, Luckenwalde, Germany) subsequently. Claudins were detected using a Zeiss 710 confocal microscope (Zeiss, Oberkochen, Germany). Control (ctrl) oocytes were injected with RNase-free water.

### 2.9. Paired Oocyte Assay with CBD Treatment: Contact Area Monitoring

To prepare the oocytes for the paired oocyte assay, the oocyte vitelline membranes were carefully mechanically removed under a binocular microscope with two fine forceps. Adding mannitol to ORi caused hypertonic cell shrinking and enabled it to grip the vitelline membrane. Next, two oocytes each heterologously expressing claudin-4, claudin-5, and claudin-18.2 (CLDN4/5/18) were pairwise attached in one well of a 24-well plate (combination: CLDN4/5/18 + CLDN4/5/18), respectively. This procedure resulted in a direct contact of plasma membranes of two single cells expressing gastric claudins for the proper assembly of TJ complexes. The wells were either filled with 2 mL ORi (pH 7.4) without CBD (ctrl), with 20 µM CBD, or with 40 µM CBD. The well plate was stored for 48 h at 16 °C.

After 24 h and 48 h, the well plate was placed under the microscope (DMI6000 B Microscope, LAS AF software Leica Microsystems, Wetzlar, Germany), and the diameter of the cell contact area was measured via bright field microscopy. Oocyte vitality was visually evaluated after the last data acquisition by assessing the oocyte morphology and cohesion under the binocular microscope.

For data evaluation, the contact length (μm) was calculated into circle contact areas (μm^2^) by using the circle formula A = π∙r^2^. Then, controls were set to 100%.

### 2.10. Double Orbital Challenge Assay

To examine the claudin interaction in more detail, the contact strength was quantified by Double Orbital Challenge (DOC) analysis, which is a standardized adhesion assay. The oocyte preparatory steps were performed as described above. To this end, two oocytes expressing claudin-4, claudin-5, and claudin-18.2 (combination: CLDN4/5/18 + CLDN4/5/18) were paired in the middle of one well of a 24-well plate as described above. Each well was filled with 1 mL ORi (pH 7.4) without CBD (ctrl), with 20 µM CBD, or with 40 µM CBD, respectively. The well plate was incubated for 24 h at 16 °C.

**Figure 1:**
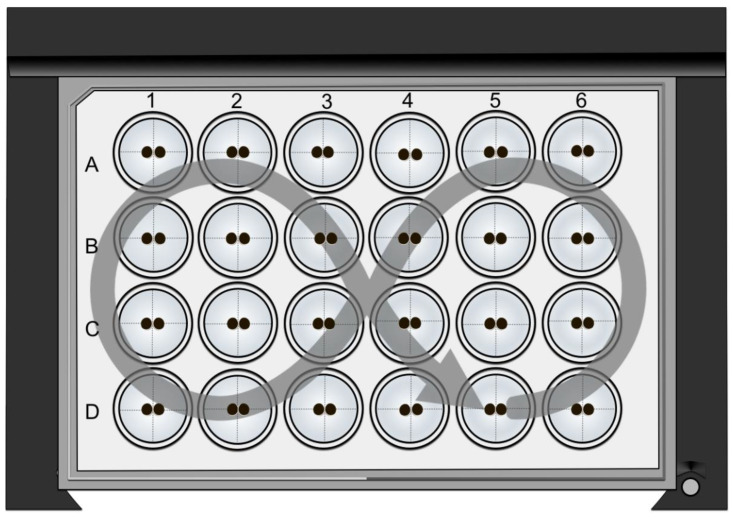
Schematic depiction of the Double Orbital Challenge (DOC), a standardized adhesion assay. The DOC was carried out with oocyte pairs placed in a 24-well plate for 120 s using a plate reader to apply constant shear stress (lettering of supplier; arrow: direction of double orbital shaking treatment).

Subsequently, the 24-well plate was placed in a Perkin Elmer Enspire (PerkinElmer EnSpire Multimode Plate Reader, Waltham, MA, USA). The shaking diameter was 1 mm, and a constant double orbital shaking treatment was adjusted for 120 s and 200 rpm for a standardized mechanical adhesion challenge representing a constant application of shear stress (Figure 1). To quantify the contact strength, the contact length was measured via bright field microscopy (DMI6000 B Microscope, LAS AF software Leica Microsystems, Wetzlar, Germany) directly before and after the double orbital shaking treatment, and the change in contact area (Δ contact area) was calculated in percentage.

A vitality assessment of the oocytes was carried out after data acquisition as described above (Section 2.9).

### 2.11. Statistical Analysis

Contact area size data from the paired oocyte assay are shown in means ± SEM and in % of the controls that were set to 100%. Double Orbital Challenge data are presented as box plots. Box plots are divided into the first (25 percent) and the second quartile (75 percent) as well as in whiskers (10th and 90th percentile). N describes the number of animals and n describes the number of experiments/oocyte pairs. Statistical analysis was carried out with JMP Pro 16.0.0 (SAS Institute Inc., Cary, NC, USA). A Shapiro–Wilk test was used to test normal distribution. The not normally distributed data were evaluated by a Kruskal–Wallis test followed by a Dunn–Bonferroni correction. Values of *p* < 0.05 (presented as * *p* < 0.05) were statistically significant.

## 3. Results

### 3.1. Expression of Claudins in the Stomach

**Figure 2:**
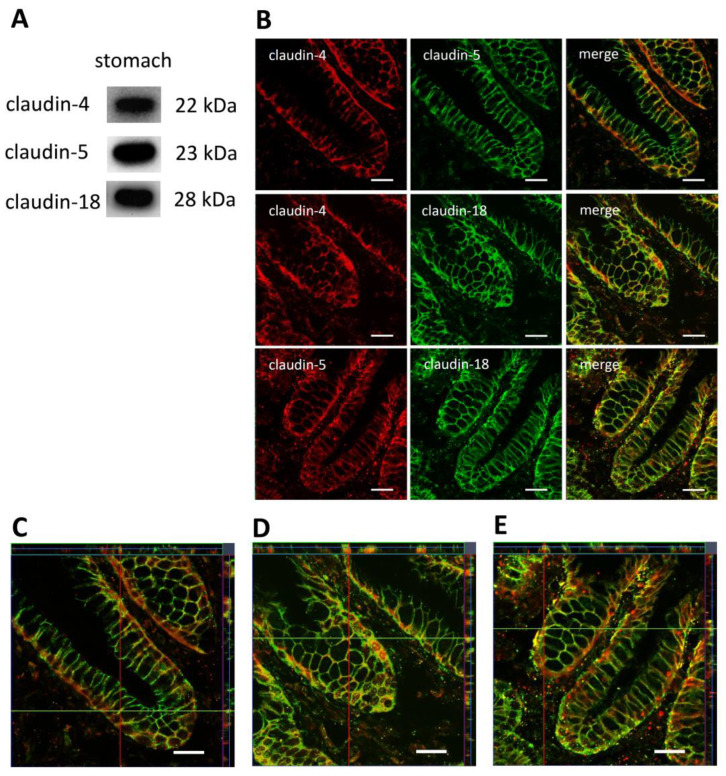
(**A**) Immunoblots of claudin-4, -5, -18 expression in porcine gastric tissue. (**B**) Localization of claudin-4, -5, -18 by confocal laser scanning immunofluorescence microscopy and (**C**–**E**) z-stacks of the respective claudin combinations; location of sections indicated by horizontal green and vertical red lines, respectively. Representative images (scale bars: 20 μm).

Porcine tissue samples were stained with specific antibodies raised against claudin-4, claudin-5, and claudin-18, and immunoblots of the gastric tissue preparation were performed. Immunoblot lane signals showed the expected sizes of 22 kDa for claudin-4, 23 kDa for claudin-5, and 28 kDa for claudin-18, confirming that all claudins can be detected in native gastric epithelium (Figure 2).

By using confocal laser scanning microscopy, claudin-4, -5, and -18 were localized in porcine stomach tissue.

Immunofluorescence images of the fundus show longitudinal sections of the *glandulae gastrica propriae*.

All claudins showed strong specific signals. Claudin-4 in red is irregularly localized compared to claudin-5 and -18 and is in the fundus mainly concentrated at the base of the cells of the *glandulae gastrica propriae*. Claudin-5 in red and -18 in green showed a uniform paracellular signal that leads to a cell network (Figure 2B). The merged images give an overview of the protein fragment’s orientation. In all co-localization combinations—claudin-4 and -5, claudin-4 and -18 as well as claudin-5 and -18—strong and clear punctual overlapping signals were detected (yellow, Figure 2B).

To obtain a more detailed overview of the localization and co-localization of the three claudins in the stomach, z-stacks were performed. All claudins were detectable in apicolateral tight junction complexes with additional localization in subjunctional areas. Yellow signals confirm, also in the z plane, a co-localization of every claudin combination (Figure 2C–E).

### 3.2. Heterologous Expression of Three Gastric Claudins in the Oocyte Membrane

Human claudin-4, -5, and -18 cRNA were injected into *Xenopus laevis* oocytes, and after three days, the expression was ensured via immunoblots and immunohistochemistry by staining oocyte tissue with specific antibodies for the gastric claudins.

**Figure 3:**
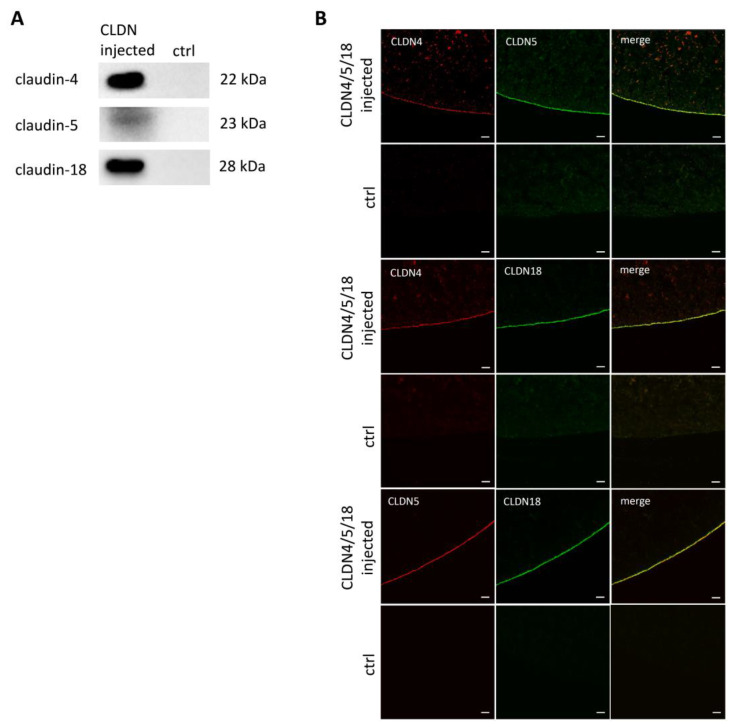
(**A**) Immunoblots of heterologous co-expression of claudin-4, claudin-5, and claudin-18.2 and (**B**) confocal laser scanning immunofluorescence microscopy to locate protein accumulation within the oocyte membrane. Representative images (scale bars: 20 μm).

Immunoblots of oocyte membrane fractions show specific lanes of every claudin in the predicted size. However, in water-injected control oocytes, no signals were detected. Thus, for the first time, the heterologous co-expression of the three human claudins claudin-4, -5 and-18 was proven (Figure 3A).

To ensure and visualize the integration of the gastric claudins into the oocyte membrane, immunohistochemistry was performed (Figure 3B).

The images show that claudin-4, -5, and 18.2 were co-expressed in the oocyte membrane of one oocyte. Claudin-4 in red predominates in the membrane, but some protein fragments can also be seen in subjunctional compartments, whereas claudin-5 in red and -18 in green are more exclusively located in the oocyte membrane. The merged images show a strong yellow signal which is not just punctual and appears mostly as a continuous line. Thus, the claudins in every combination are co-localized. In contrast, in water-injected control oocytes, no specific protein signals can be seen in the oocyte membrane.

### 3.3. Paired Oocyte Assay for Contact Area Analysis

To investigate the *trans*-interaction of claudin-4, -5, and -18 depending on 20 µM and 40 µM CBD, the contact area of two paired oocytes expressing the human gastric proteins after 24 h and 48 h were analyzed. The controls, claudin-4, -5, and -18 expressing oocyte pairs that are not incubated with CBD, were set to 100%.

**Figure 4:**
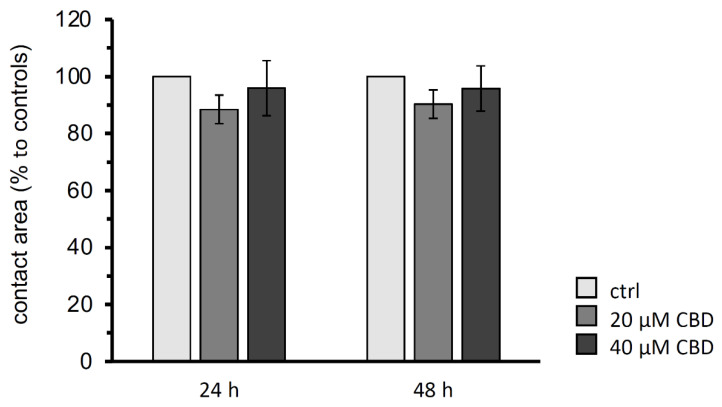
Contact areas size analysis of the paired oocyte assay after 24 h and 48 h. CBD revealed no changes in contact areas of claudin 4/5/18 expressing oocyte pairs (CLDN4/5/18 + CLDN4/5/18). Data are presented in mean ± SEM (N = 6, ctrl: n = 18, 20 µM CBD: n = 28, 40 µM CBD: n = 24, *p* > 0.05, Kruskal–Wallis test followed by a Dunn–Bonferroni correction).

The paired oocyte assay showed no significant effects of 20 µM or 40 µM CBD on the contact area representing the *trans*-interaction area of the three gastric claudins after 24 and 48 h (N (number of donor animals) = 6; 20 µM CBD: 24 h: *p* = 0.5752, 48 h: *p* = 0.4435, n (number of experiments/oocyte pairs = 28; 40 µM CBD: 24 h: *p* = 1.0000, 48 h: 0.9778, n = 24; Figure 4).

### 3.4. Double Orbital Challenge for Quantification of Contact Area Strength

**Figure 5:**
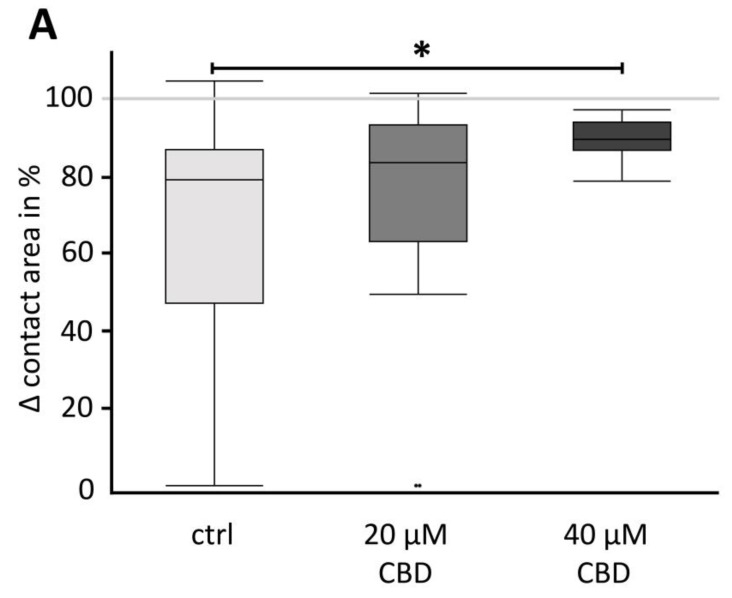

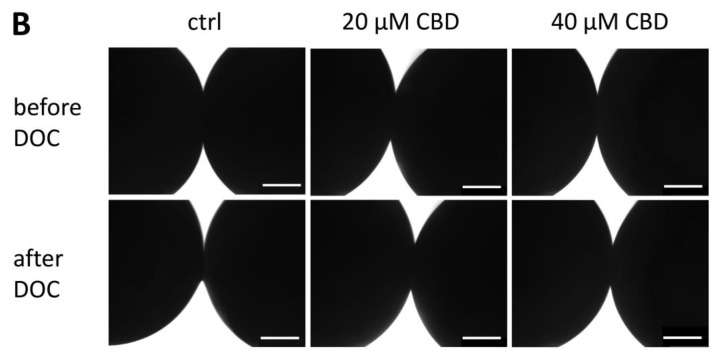
Contact strength analysis using the Double Orbital Challenge (DOC) (**A**) in box plots and (**B**) visualization of paired oocytes (CLDN4/5/18 + CLDN4/5/18) by transmitted light optical microscopy before and after carrying out DOC. The Δ contact area after incubation with 40 µM CBD was significantly larger than in control oocytes (N = 3, n = 11, * *p* = 0.0472, Kruskal–Wallis test followed by a Dunn–Bonferroni correction). In contrast, the contact area difference of the oocyte pairs incubated with 20 µM CBD and ctrl was not significantly altered. Representative images (scale bars = 100 µm).

To analyze the *trans*-interaction in more detail, the contact area strength of the oocyte combination CLDN4/5/18 + CLDN4/5/18 was quantified. For this, oocyte pairs were placed in a 24-well plate with ORi and were incubated for 24 h with 20 µM or 40 µM CBD or without CBD (Figure 1). After 24 h, CLDN4/5/18 + CLDN4/5/18 pairs in 40 µM CBD maintained a 23.6 % higher contact area than oocyte pairs without CBD (* *p* < 0.05). In contrast, 20 µM CBD did not affect the contact strength significantly. Therefore, the higher concentration of CBD of 40 µM has a strengthening effect due to the stronger cohesion of the contact area (N = 3; ctrl: 65.97 ± 7.93 %, n = 16; 20 µM CBD: 73.75 ± 8.45 %, *p* = 0.4442, n = 15; 40 µM CBD: 89.56 ± 1.78 %, *p* = 0.0472, n = 11; Figure 5A,B).

## 4. Discussion

TJs and claudin integrity play a key role in gastrointestinal barriers. Claudin-4, -5, and -18 are critical proteins of gastric TJs, sealing the paracellular between two neighboring epithelial cells of the gastric mucosa, enclosing luminal factors, and promoting gastric functions, respectively [19,20,21,22]. The integrity of TJs determine the tightness of a barrier, and these properties are dependent on the specific claudin combination [26]. The gastric epithelium is considered to have greater tightness [18]. Therefore, claudin-4, -5, -18.2 strongly contribute to this strengthening property of the gastric epithelial cells. The sealing functions of the single proteins have been reported in different studies. In follicle–associated epithelia of porcine Peyer’s patches, claudin-4 strengthens the sealing of the paracellular pathway and affects the selectivity [27]. Claudin-5 showed also a sealing effect in Caco-2 cell monolayers [28]. Moreover, Claudin-5 is expressed in the blood–brain barrier and provides strong sealing properties [29]. The isoform 2 of claudin-18 is exclusively expressed in the stomach, and its TJ assembly is characterized by tightly packed parallel arrays of TJ strands [30]. Many studies have shown that the deletion of claudin-18 causes an increased paracellular H^+^ leakage in the stomach. Thus, the claudin forms closely anastomosing TJ strands and seals the paracellular barrier against luminal protons [21,30,31,32].

The expression of these claudins could be detected in the stomach by both immunoblotting and immunohistochemistry. In accordance with the literature, claudin-4 signals were localized in tight junction complexes, showing that the porcine model can be employed as a reference for further studies as well [19,20]. In accordance, we also observed a strong expression of claudin-4 in base cells of the fundus and evenly distributed signals for claudin-5 and claudin-18 in all preparations of the *glandulae gastrica propriae*. Co-localization was confirmed by yellow signals in all pairwise combined claudin detections. This co-localization was even verified in higher resolution in z-stacks for every claudin combination (claudin-4 + claudin-5, claudin-4 + claudin-18, claudin-5 + claudin-18).

Our study aimed to investigate the direct effects of cannabidiol (CBD) on human claudin-4, -5, and -18 clusters in *Xenopus* oocytes that were used as a heterologous expression and interaction model. Via co-expressed claudin cluster pairing, we provided a standardized contact area for cell–cell and therefore *trans*-claudin interaction. Accordingly, interaction challenge experiments resulted in quantitative data regarding the *trans*-interaction size and strength.

The prerequisite for carrying out this analysis was the successful co-expression of the three claudins in the oocyte membrane. We detected specific signals of the human claudins using immunoblots of membrane fractions of the co-injected oocytes. Furthermore, immunohistochemistry stainings show the incorporation into the oocyte membrane and indicate a co-localization by overlaying signals of all stained combinations (yellow, claudin-4 + claudin-5, claudin-4 + claudin-18, claudin-5 + claudin-18). We therefore achieved for the first time a successful expression of the three gastric claudins after three days of injection. Recently, the two gastric claudins -4 and -18.2 have already been reported to be successfully co-expressed in the oocyte membrane [13]. 

Cannabinoids derived from herbal *Cannabis spec.* are attracting increasing attention for anti-inflammatory and immunomodulating properties, and specific effects are still under-investigated. Currently, the research on individual components of the plant is ongoing. The most well-known secondary plant extract is cannabidiol (CBD) [33,34,35,36]. In a latest study, CBD’s effects on GABAergic neurotransmission were investigated in *Xenopus* oocytes after microtransplanting human brain membranes [37]. However, information regarding direct effects including effects on the gastric epithelium is scarce. A murine study has shown that CBD is associated with anti-inflammatory and anti-ulcer effects in the gastrointestinal tract and can suppress gastrin production and gastric acid secretion [38]. Furthermore, a human study showed that cannabis consumption reduces alcohol-associated gastritis. Thus, cannabis might have modulatory effects on the gastric mucosa [39]. The results of our CBD experiments show the direct effect of CBD on claudin-4, -5, and -18 on intercellular interaction. We were able to show that CBD has no effects after 24 h and 48 h on the contact area, but it increases the strength of the contact area. Our results suggest that CBD may have a strengthening modulatory effect on the TJ assembly of claudins after 24 h. In addition, our group showed that CBD in IPEC J2 cells in the concentration of 40 μM has a decreasing effect on the paracellular permeability of [^3^H]-D-Mannitol after 48 h, also indicating a barrier-strengthening effect. Furthermore, the claudin-4 expression was markedly increased [25]. However, a general difference and prerequisite of the current study is a lack of endogenous CB receptors in the oocytes, allowing the study of immediate effects on tight junction integrity [40].

The measurements of the contact strength were carried out with a newly established adhesion assay, DOC. The improved method for testing the contact strength allows us to carry out an automated and standardized challenge with better reproducibility. This has the advantage of applying constant shear stress to 24 oocyte pairs at the same time.

To ensure constant oocyte quality, we were investigating experimental measurements to a maximum of 48 h after starting interaction experiments with paired oocytes. This corresponds to a post-harvest time of 6 days, which is in accordance with the specified oocyte vitality period of 10 to 15 days [41]. In addition to the time scheme, oocyte quality was preserved by using an antibiotic-containing oocyte Ringer and regular vitality checks. In accordance with this time frame, the experimental setup of the interaction experiments and therefore compound effect screening is currently limited to 48 h.

Overall, the experimental design of our established two-cell model is constantly evolving to provide unique insights into the functional analysis of proteins using the oocyte as a heterologous expression model. Additionally, the impact of single claudins on TJ assembly and the functional contribution may be further analyzed in detail.

The results of the functional studies using the *Xenopus* oocytes as a heterologous expression model show that the size of the contact area, the size of the *trans*-interaction area, is not changed by CBD. In contrast, the newly established DOC experiment revealed an increased contact strength after 24 h incubation with CBD. CBD could directly affect the claudin *trans*-interaction and therefore increase the described sealing properties. This contributes to supportive gastric barrier effects and adds to a multilevel analysis of cannabinoid effects.

With this study, we have expanded our established stomach model [13]. The heterologous expression of human gastric-specific claudins in the *Xenopus laevis* oocyte allows the direct examination of secondary plant compounds as well as other extracellular factors to the gastric mucosa and provides new possibilities for *trans*-interaction and TJ integrity analyses of barrier-modulating mechanisms.

## 5. Conclusions

In conclusion, we successfully established the heterologous co-expression of three human gastric transmembrane TJ proteins, claudin-4, -5, and -18.2 in *Xenopus laevis* oocytes. The newly developed Double Orbital Challenge assay employing paired oocytes was performed to analyze the direct effects of CBD on the gastric TJ integrity. CBD revealed an increased contact strength after 24 h. Therefore, for the first time, a direct effect of CBD on protein–protein *trans*-interaction was observed.

## Data Availability

Data are contained within the article. The datasets analyzed during the current study are available from the corresponding author upon reasonable request.
